# ‘Utilitarianism for animals: deontology for people’ and the doing/allowing distinction

**DOI:** 10.1007/s11098-021-01745-7

**Published:** 2021-10-28

**Authors:** Fiona Woollard

**Affiliations:** University of Southampton, Southampton, UK

**Keywords:** Utilitarianism for Animals, Hybrid Approaches, Animal Ethics, Doctrine of Doing and Allowing, Deontology

## Abstract

It is tempting to think that zebras, goats, lions, and similar animals matter morally, but not in quite the same way people do. This might lead us to adopt a hybrid view of animal ethics such as ‘Utilitarianism for Animals; Deontology for People’. One of the core commitments of deontology is the Doctrine of Doing and Allowing (DDA): the view that doing harm is harder to justify than allowing harm. I explore how this core tenant of deontology applies to non-person, non-human animals and whether hybrid views of animal ethics can accept it. In doing so, I aim to do three things. First, to show that my defence of the DDA can solve a problem surrounding our duties to wild animals, while making only minimal claims about animal moral status. Second, to offer an argument that for many non-person, non-human animals, we should recognise deontological constraints on their treatment, but also see those constraints as importantly different from the constraints against doing harm to persons. Third, to get clearer on how we should understand Utilitarianism for Animals and Nozickian hybrid approaches to animal ethics.

## Introduction

If anything at all is obvious, it is obvious that it is wrong to torture a kitten for fun. It is also, I think, obvious that the main reason that it is wrong for me to torture a kitten is because it would be bad *for the kitten*. Kittens–and many other animals–are morally considerable in their own right and for their own sake. They have moral status. Nonetheless, it may seem equally obvious that these animals do not have quite the same moral status as you or I. There are lots of things that it would be clearly impermissible to do to people which many think permissible when it comes to animals like kittens: for example, many think it permissible to keep kittens as pets, so long as this does not interfere with their wellbeing.

This may lead us to adopt a hybrid view of animal ethics. A hybrid view is any view according to which different classes of individuals ought to be treated according to different principles. Thinking about examples like the one above, we might be led to think that what really matters when it comes to animals like kittens is overall welfare. This might lead us to the most well-known hybrid view of animal ethics, pithily described by Robert Nozick as “Utilitarianism for animals, Kantianism for people” (Nozick, 1974, 39). Given that many deontologists don’t see themselves as following in Kant’s footsteps, I rephrase this as: “Utilitarianism for Animals, Deontology for People.”

One core commitment of deontology is the Doctrine of Doing and Allowing (DDA): doing harm is harder to justify than allowing harm. This paper explores how this core tenant of deontology applies to non-person, non-human animals and the implications of considering the DDA for Nozickian hybrid views of animal ethics.

To work out whether and how the DDA applies to non-person, non-human animals, we need to think about what the DDA is and why it should be accepted for people. We need to look at the details of a proposed defence of the DDA. I focus on my own defence of the DDA (Woollard, 2015). However, my discussion has wider significance. I end the paper by identifying conditions for a defence of the DDA to be ‘structurally similar’ to my account and the implications of this discussion for any such account.

I described the DDA above as the claim that doing harm is harder to justify than allowing harm. This means that considerations that may make it permissible to *allow* a given harm are not enough to make it permissible to *do* that same harm. One way to justify countenancing harm is by appeal to *avoidance costs to the agent*: what it would cost the agent to avoid countenancing the harm. Suppose Ann spots Victor drowning, but does not stop and help because she is on her way to hospital and will lose her arm if she delays. Ann allows harm to Victor. It is permissible for Ann to allow Victor to die rather than lose her arm. However, it would not be permissible for Ann to push Victor into the deadly water (doing harm to him) if this were the only way for her to get to hospital in time. In this pair of cases, if Ann were to try to justify her behaviour, she would appeal to the *avoidance cost to the agent*. In both cases, the avoidance cost is the same: the loss of an arm. This avoidance cost is enough to justify letting Victor die but not killing Victor. Doing harm to persons is *harder to justify by appeal to the avoidance cost to the agent* than allowing harm to persons.

A second common way to justify countenancing harm is by appeal to the greater good. Suppose that Alice is driving five mortally wounded strangers to the hospital. We might think it is permissible for Alice to refuse to stop and save Victor from drowning in order to get the strangers to hospital on time, but not permissible for Alice to push Victor into the deadly water to save the strangers. In this pair of cases, if Alice were to justify her behaviour she would appeal to the greater good: only one death instead of five. Saving five people is enough to justify allowing one to die but not to justify killing them. Doing harm to persons is *harder to justify by appeal to the greater good* than allowing harm to persons.

I show that my defence of the DDA straightforwardly implies that there is at least some moral difference between doing harm to non-human, non-person animals and allowing harm to such animals. My defence of the DDA straightforwardly implies that doing harm to such animals is harder to justify *by appeal to the avoidance cost to the agent* than allowing harm to such animals. I show that this result is important: it means that we can avoid counterintuitive implications about our duties to save wild animals without appealing to more than very minimal claims about animal moral status. However, this very straightforward application of my account does not give us constraints against doing harm to non-person, non-human animals. It does not leave doing harm to such animals harder to justify *by appeal to the greater good* than allowing harm to such animals.

I then explore whether we should endorse constraints against doing harm to such animals. On my account, we should endorse constraints against doing harm for a given animal if and only if that animal’s body belongs to them in a morally significant way. I give an argument that at least some non-person, non-human animals’ bodies belong to them, but that there are important differences in the way in which a person’s body belongs to them and the way in which such an animal’s body belongs to them. If my account of these differences is correct, we should recognise constraints against doing harm to non-human, non-person animals, but understand those constraints as importantly different from–and in many ways weaker than—the constraints against doing harm to people.

Finally, I explore what consideration of the DDA implies for Utilitarianism for Animals. I show that my discussion of the straightforward application of my DDA defence suggests that we should reject Nozick’s original understanding of Utilitarianism for Animals. I propose a way of understanding Act-Utilitarianism for Animals which leaves the theory more plausible. Nonetheless, if I am right that many non-person, non-human animals’ bodies belong to them and thus that there are constraints against doing harm to them, we should reject even this form of Act-Utilitarianism for Animals.

When we discuss “Utilitarianism for Animals, Deontology for People”, my inclination is to take the class of people to include *both* all embodied persons *and* all human children and adults of any cognitive ability.

I can sketch an argument for the inclusion of all embodied persons. As my defence of the DDA appeals to features of embodied persons, rather than of humans in particular, my DDA ‘for people’ applies to any non-human animal persons. More broadly, any Deontology for People that is defended by appeal to features of persons in general rather than humans in particular should also apply to non-human persons.

I cannot in this paper offer a defence for the inclusion as people of all human children and adults of any cognitive ability. Whether there exist any human children or adults who are not persons and if so, how they should be treated is clearly a key issue for Nozickian hybrid views. It is also an extremely complex one and sensitive issue–which deserves more space than I can give it in this paper.^1^ In any case, I do not take anything I say in this paper to warrant the exclusion of any humans from the category of people protected by deontological principles. This paper focuses on the application of the DDA to animals that are neither persons nor humans. In what follows, I will refer to these as N-P&N–H-animals.^2^, ^3^

## My defence of the DDA

I defend the Doctrine of Doing and Allowing by arguing that it is necessary for anything to genuinely belong to anyone, even their own body. Genuine belonging requires protection against being imposed on. This includes protection against causal imposition (where the behaviour of others intrudes on what belongs to me, making substantial changes to what belongs to me without my consent). This means that for my body and other resources to genuinely belong to me there need to be constraints against doing harm (Woollard, 2013, 2015).

But protection against causal imposition is not enough. A resource does not genuinely belong to me if I can be arbitrarily required to put it at the use of others. For my body and resources to genuinely belong to me, I also need protection against normative imposition (where the needs of others intrude on what belongs to me, requiring me to put what belongs to me at the use of another). Protection against normative imposition requires permissions to allow harm. (Woollard, 2013, 2015).

On this view the Doctrine of Doing and Allowing is made up of two elements: (1) Constraints against doing harm which respect that the potential victim’s body and other resources belong to them; (2) Permissions to allow harm which respect that the agent’s body and other resources belong to them. There is a third element in the background: (3) Reasons to reduce harm and to help others which respond to a general concern for the good of creatures.

One way of recognising the third element is to note that the constraints and permissions provided by the Doctrine of Doing and Allowing are not absolute. Almost no one thinks that it is always impermissible to do harm, no matter how small the harm and how large the cost of avoidance. Almost no one thinks that it is always permissible to allow harm, no matter how large the harm and how small the cost of avoidance. Any plausible version of the Doctrine of Doing and Allowing provides strong protection against very harmful imposition and weaker protection against less harmful impositions. This is why the Doctrine of Doing and Allowing can be understood as the claim that, other things equal, doing harm is harder to justify than allowing harm. This version of the Doctrine of Doing and Allowing recognises that our authority over what belongs to us does not always override concern for the good of creatures.

Moreover, protection against normative imposition only makes sense under the assumption of a background of standing reasons to reduce harm and to help others. The point of the permissions to allow harm is to limit the demands that the needs of others make on the agent, preventing those demands from undermining her authority over what belongs to her. Without a background in which the needs of others do place demands on us, there is nothing to limit.

## Application to animals: a straightforward answer

So, on this view, the Doctrine of Doing and Allowing is best understood as a principle governing the interactions of these three morally relevant factors: the authority of the potential victim over their body; the authority of the agent over their body and the good of creatures. But, aside from a short footnote (Woollard, 2015, footnote 17, p 108), my earlier work defending the Doctrine of Doing and Allowing focuses on cases in which both the agent and the potential victim(s) of harm are people. What happens when we consider the ethics of our interactions with N-P&N–H-animals? Does this defence of the Doctrine of Doing and Allowing extend to cases where a person could countenance (either do or allow) harm to such animals?

It follows straightforwardly from my account that the Doctrine of Doing and Allowing at least partially applies to persons’ countenancing harm to any sentient animal. If we have an agent who is a person and a sentient animal potential victim, then we have at least two of the three elements of the Doctrine of Doing and Allowing. First, the agent’s body and other resources belong to them. They need protection against normative imposition. Second, the animal potential victim is the type of creature that can be harmed or benefitted in a morally relevant way. We have standing reasons based on the good of creatures to reduce harm to the animal and to help them.

These two elements are enough to give rise to a moral distinction between doing harm and allowing harm to any sentient animals–including N–P&N–H-animals. Agents have standing reasons based on the good of creatures not to allow harm to such animals. However, requirements to avoid allowing harm are limited. Requirements to avoid allowing harm normatively impose on the agent: they place the agent’s body and other resources at the use of another. Permissions to allow harm are needed to respect that the agent’s body and other resources belong to them. Agents also have standing reasons based on the good of creatures not to do harm to N–P&N–H-animals. Requirements to avoid doing harm do not (as such) impose on the agent. So requirements to avoid doing harm are not limited in the same way by the need to respect that the agent’s body and other resources belong to them. Doing harm to a N–P&N–H-animal is harder to justify than allowing harm to such an animal.

## Hills’ Problem

This result helps avoids a serious problem in animal ethics. Alison Hills argues that once we take wild animals into account, Universal Utilitarianism–Utilitarianism for *both* people and other animals—becomes implausibly demanding (Hills, 2010).^4^ There are currently millions of wild animals that are at risk of starving, suffering from diseases, being maimed by accident or by attempts at predation. Utilitarianism seems to demand that we intervene, if not to save the wild animals, at least to reduce their suffering.^5^ As Hills argues, wild animals present a permanent emergency situation which, unlike the human suffering caused by global poverty, cannot be blamed on unjust social structures. “It is difficult, and probably impossible, to devise any kind of political system or institution to solve the problems of animals without our constant supervision and intervention. Our duties to animals are more demanding, not in the sense that they are more urgent than our obligations to humans (they are not), but in the sense that it is even more difficult to discharge them. The impact on our life will be almost as great, and, realistically, will never be over. Our personal projects will be in constant conflict with these demands: our integrity will be permanently compromised” (Hills, 2010, 237).

Utilitarians may be tempted to reply that they can easily avoid this result by discounting N–P&N–H-animal pain. Hills shows that such a response is not tenable, for it commits the Utilitarian to another, equally undesirable, result. The problem, as Hills notes, is that Utilitarians do not recognise any moral distinction between doing and allowing harm. Suppose that, for example, the Utilitarian wants to show that I am not required to give up my career to prevent zebras being devoured alive by lions. They argue that although zebra pain matters morally, it doesn’t matter as much as my career. I am permitted to allow hundreds of zebras to be eaten alive in order to purse my personal projects. Because the Utilitarian doesn’t recognise a distinction between doing and allowing harm, they have to hold that any potential cost that would justify allowing a given amount of N–P&N–H-animal pain will also justify bringing about that same amount of N–P&N–H-animal pain. So if they endorse (A) that I am permitted to allow hundreds of zebras to be eaten alive in order to purse my personal projects they must also endorse (B) I am permitted to bestow the same kind of pain on hundreds of zebras if this is necessary for me to pursue my personal projects. But most Utilitarians do not want to accept (B).^6^ There are similar problems for other moves that the Utilitarian might want to make. If they adopt a satisficing or scalar Utilitarianism which implies (A’) that preventing the zebras’ pain is morally optimal but not required, then they end up forced to also endorse (B’) that it is optimal but not required to avoid inflicting hideous pain on zebras in order to pursue my personal projects. Again, most Utilitarians do not want to accept (B’). This objection to Utilitarianism is Hills’ Problem.

Hills’ Problem is a problem for any account which does not recognise a distinction between doing harm to N–P&N–H-animals and allowing harm to such animals. In fact, Hills’ Problem may be even *more* pressing for hybrid theories which recognise a distinction between doing and allowing harm to people but not to N–P&N–H-animals–for such theories would have to choose between the unacceptable (B), (B’) etc. claims and the highly counterintuitive implication that our duties to wild animals are *more* demanding than our duties to people.^7^

So my defence of the DDA is helpful. It allows us to recognise a moral difference between doing harm to N–P&N–H-animals and allowing harm to such animals while making only fairly minimal commitments concerning N–P&N–H-animal status: all that is needed is the claim that (a) the agent’s body and other resources belong to them and (b) the N–P&N–H-animal potential victim is the type of creature that can be harmed or benefitted in a morally relevant way. We can thus avoid Hills’ Problem when it comes to animals without making controversial claims about N–P&N–H-animal moral status.^8^

## Minimal versus full-blown DDA^9^

I argued above that my defence of the DDA can be used to recognise a moral difference between doing harm to N–P&N–H-animals and allowing harm to such animals while making only fairly minimal commitments concerning N–P&N–H-animal status. This argument doesn’t give rise to quite the same DDA that applies to persons.

Remember that my DDA for persons is made up of three elements: (1) Constraints against doing harm which respect that the potential victim’s body and other resources belong to them; (2) Permissions to allow harm which respect that the agent’s body and other resources belong to them; (3) Reasons to reduce harm and to help others which respond to a general concern for the good of creatures.

Appeal to the agent’s authority over their body and resources and the moral relevance of harms and benefits to the animal only provide the second and third of these elements. Let’s call this version of the Doctrine of Doing and Allowing produced by the second and third elements of the DDA, the Minimal Doctrine of Doing and Allowing.

The Minimal DDA is a Doctrine of Doing and Allowing in an important way: it implies that doing harm to a N–P&N–H-animal is harder to justify than allowing harm to such an animal. However, it does not include the first element of the Doctrine of Doing and Allowing: constraints against doing harm springing from the need to respect that the potential victim’s body and resources belong to them. This means it falls short of the version that applies to persons in some important ways. First, although there are reasons not to do harm to N–P&N–H-animals based on a standing concern for the good of creatures, these are much weaker than the constraints against doing harm to persons based on respect for a person’s authority over their body. So, it may be possible to justify doing harm to N–P&N–H-animals based on comparatively weak costs to the agent of avoiding doing harm.^10^ Secondly, the moral difference between doing and allowing harm appeals purely to the need for permissions for the agent to allow harm. Thus, while it implies that doing harm to N–P&N–H-animals is harder to justify *by appeal to costs to the agent* than allowing harm to such animals, it does not imply that doing harm to N–P&N–H-animals is harder to justify *by appeal to the greater good* than allowing harm to such animals. The Minimal Doctrine of Doing and Allowing is compatible with the permissibility of killing one zebra to save two zebras.

I will call the version that applies to persons and is made up of all three elements, the Full-Blown DDA.

**Table:** 

	Minimal DDA for Xs	Full-Blown DDA for Xs
Element 1: Constraints against doing harm to X	No	Yes
Element 2: Permissions to allow harm to X	Yes	Yes
Element 3: General concern for the good of creatures	Yes	Yes
Doing harm to Xs is harder to justify by appeal to cost to the agent	Yes–but doing harm may be justifiable based on comparatively weak cost to agent	Yes
Doing harm to Xs is harder to justify by appeal to the greater good	No. Permissible to kill one X to save two Xs	Yes. Impermissible to kill one X to save two Xs

## Minimal or full-blown DDA for N–P&N–H-animals?

On my account, a Full-Blown DDA for (at least some) N–P&N–H-animals can be defended if and only if we can hold that (at least some) N–P&N–H-animals’ bodies genuinely belong to them, in a way that gives rise to constraints on imposing on their bodies.

In my defence of the DDA for persons, I call the claim that a person’s body genuinely belongs to them, the Body Claim (Woollard, 2015, 187). Let us now distinguish between the Body Claim for Persons (each person’s body genuinely belongs to them) and the Body Claim for N–P&N–H-Animals (for some N–P&N–H-animals, the animal’s body genuinely belongs to them). Before exploring the defences of the Body Claim for Persons and the Body Claim for N–P&N–H-Animals, I will explain what it means to say that a creature’s body genuinely belongs to them. I will also address concerns about whether these claims make sense: both concerns about whether it makes sense in general to claim that someone’s body belongs to them and whether it makes sense to claim that something belongs to a N–P&N–H-animal.

Remember here that I focus on N–P&N–H-animals: animals that are neither persons nor humans. If some non-human animals such as great apes and dolphins are persons, then they will be covered by my original defence of the Body Claim for Persons.

My understanding of what it is for a person’s body to belong to them draws on Jeremy Waldron’s account of the concept of private property. As I previously explained:

[According to Waldron] A property system provides an answer to the problem of allocation: the problem of determining peacefully and reasonably predictably who is to have access to which resources for what purposes and when (Waldron, 1988, 32). In a (pure) private property system, the allocation problem is solved by assigning resources to individuals. ‘Imagine that the material resources available for use in a society have been divided into discrete parcels (call each parcel an object) and that each object has the name of an individual member of the society attached to it... In a private property system, a rule is laid down that, in the case of each object, the individual person whose name is attached to that object is to determine how the object shall be used and by whom’ (Waldron, 1988, 38-9). Waldron’s image of individual names attached to parcels of resources illustrates nicely what I mean when I talk about a resource belonging to a person. A resource belongs to a person when there is a relationship between the person and the resource in virtue of which the resource is allocated to that person: it is his or hers in a way that gives the person a privileged status over the use of that resource. This privileged status gives the person a prima facie authority to make decisions over what happens to that resource based primarily on his or her own interests and desires. The Body Claim states that each person’s body must belong to him or her. It must be recognized as his or hers in a way that gives the person a prima facie authority to make decisions over what happens to it based on his or her own interests and desires (Woollard, 2015, 187-186).

This way of explaining the Body Claim responds to a common concern about whether it makes sense to speak of someone’s body belonging to them. According to this concern, because my relationship to my body is so special, it just does not make sense to describe my body as belonging to me. But if we think of belonging in terms of the allocation of a resource, we see that this objection is misguided. However special my relationship to my body is, my body is still a resource. As I argue elsewhere:

The state of my body, and the things that it does and that are done to it, can affect the welfare of myself and others. It can be used to achieve ends—either my ends or the ends of others. Moreover, either I or another may take my body being in a certain state as an end. Finally, others can affect it in ways that diminish its usefulness or its value. We thus face the allocation problem with respect to my body: on what basis should decisions be made about what happens to it? A sensible answer to this question (indeed I think the only plausible answer) is that I should have prima facie authority to make decisions about what happens to my body on the basis of my interests and desires. If this answer makes sense, the claim that my body belongs to me makes sense (Woollard, 2015, 188).

The bodies of N–P&N–H-animals are also resources. It makes sense to ask on what basis decisions about the use of those resources should be made. So just as it makes sense to speak of a person’s body belonging to someone, it makes sense to speak of the bodies of N–P&N–H-animals belonging to someone. However, in the case of the N–P&N–H-Animal Body Claim, there is an additional concern. Suppose we agree that the bodies of N-P&N–H-animals are the kinds of things that can belong to someone. Are N–P&N–H-animals the kinds of creatures to which things can belong?

My understanding of belonging for persons has two parts: first, that the person has the authority to decide what happens to the resource; second, that they are entitled to do so based on their own interests and desires. Does it make sense to speak of a N–P&N–H-animal having authority to decide what happens to their body? Shelly Kagan argues that many such animals have at least some autonomy: “Animals still make choices, after all, even if less grand than some of the choices that we make. A dog, for example, may decide between chasing its tail and chasing after a squirrel. That may be a less impressive and far reaching choice than your choice of career, but it is still a choice for all that. Nor should we think autonomy is only involved when deliberating about one’s life as a whole: your autonomy is also made manifest when you are doing nothing more important or significant than deciding whether to have the chocolate cake or the apple pie for dessert” (Kagan, 2019, 198).^11^ Richard Healey and Angie Pepper argue that there are many areas of decision making where animals have a right of self-determination. Like Kagan, they emphasise that the human interest in autonomy applies to “mundane and quotidian exercises of our agency”—whether to engage in physical contact or play with others, what to eat and whether to go for a run. Given that many animals are able to engage in similar activities and to make informed decisions about whether to do so, they argue that animals have a similar interest in determining the course of their lives in these ways (Healey & Pepper, 2020).^12^

This reminder not to overlook the ways in which N–P&N–H-animals make decisions is important. It also seems as if such choices by N–P&N–H-animals can make a difference to the permissibility of the types of behaviour usually ruled out by deontological doctrines like the DDA. Consider Precious, a service dog who was killed after jumping in front of her owner to protect him from an alligator.^13^ Such sacrifice seems different from simply using the dog to save a human. This seems to be because the dog has in some way exercised her authority over her own body.^14^

There may still be cases where the decisions about what should happen to a N–P&N–H-animal’s body rest on considerations which they are not capable of grasping. Often these will be the cases where decisions have the most far-reaching consequences and thus seem most important. This may make someone reluctant to see a N–P&N–H-animal as having authority to make decisions about what happens to their body overall.

Luckily, we can make sense of a N–P&N–H-animal’s body belonging to them whether or not they have authority to make decisions about what happens to their body. When someone is capable of making a decision about what happens to their body, the authority to make this decision is an important part of their body genuinely belonging to them. But if they are not able to make a decision, we can understand a resource as belonging to them when what happens to the resource is to be decided on the basis of that creature’s interests and desires (Woollard, 2015, footnote 17, p. 108). Many N–P&N–H-animals have interests and desires.^15^ The Body Claim for N–P&N–H-Animals makes sense.

I make two core arguments in defence of the Body Claim for Persons. Both these arguments appeal to three features of a person’s relationship to their body that make up the body’s unique role in the person’s interaction with the world. First, the body is the locus of my agency: it is through my body that I act on the world. Second, the body is the main locus of patience: with a few possible exceptions, it is through my body that the world acts on me. Third, my body is a major locus of interest for me: what is happening to my body is a significant part of how well or badly things are going for me.

These features mean that my body is identified with me in a way no other object is. My body is the paradigm example of a resource that comes with a name already attached. In my first core defence of the Body Claim for Persons, I argue that this connection between myself and my body is vital to my understanding of myself and my relationship to the external world. Any moral code which does not give me sufficient authority over my body does not take this connection to be normatively significant. It treats as insignificant a fact that is central to my self-understanding (Woollard, 2015,193). Adoption of such a code undermines my relationships with others: by permitting others to treat me as if a significant fact about me were not true, such rules may prevent me from forming relationships with others that stand on an appropriate footing. It also undermines my self-conception, for it says that these important facts about me are not worth recognition (Woollard, 2015, 195).

My second core argument in defence of the Body Claim for Persons argues that it is necessary for a valuable kind of agency, which I call Full-Fledged Agency. “Full-Fledged agency involves not simply acting but selecting one’s own ends and adopting a settled course of action in accordance with those ends. By adopting a settled course of action, I mean setting a plan with a genuine intention of pursuing that plan to completion” (Woollard, 2015, 196). Others doing things to my body obviously interferes with my agency. Requirements to put my body at the use of others do not conflict with normal agency–they require me to do things–but they conflict with Full-Fledged Agency by preventing me from choosing my own ends: my body is co-opted to the ends of others or to a moral end of impartial goodness. They also prevent me from forming the genuine intention of pursuing a plan to completion, for they require me to hold myself ready to give over the use of my body, the locus of my agency, whenever demanded.

Do these defences of the Body Claim for Persons transfer across to the Body Claim for N-P&N–H-Animals? This is a complex question which connects to an extensive existing literature on the moral status of animals.^16^ Given the aims of the paper, I cannot give a full argument here. Instead I will outline an argument, identifying the key premises about N-P&N–H-animals which are required for my conclusion.

I think that there are two key questions for working out whether a N–P&N–H-animal has a special connection to their body which means their body belongs to them in a way that gives rise to constraints against doing harm: (a) whether (and to what extent) the animal acts, is acted upon, and has interests *in a morally significant way*; (b) whether (and to what extent) the animal acts, is acted upon, and has interests, in these morally significant ways, *as an individual*.

Imagine a non-sentient single celled organism that moves about, and is changed by its environment, and that some of these changes increase or decrease its likelihood of surviving or of reproducing. It might make sense to talk of this creature acting or being acted on, or of things going well or badly for it. Nonetheless, as there is nothing that it is like to be that creature, the agency, patience and interest it displays are semi-metaphorical and not morally significant for their own sake. Given this, it cannot be morally significant that the creature’s body is the locus of this semi-metaphorical agency, patience and interest. The single-celled organism’s body does not belong to it in a way that gives rise to constraints against doing harm because it is not the case that the organism acts, is acted upon, and has interests *in a morally significant way*.

Similarly, we can imagine creatures that act or are acted upon or have interests in morally significant ways, but only as a kind of cell of a larger collective. The ‘hive-mind’ is a common trope of science fiction: in such stories, aliens in separate bodies are shown to be connected to a single collective consciousness. Although there may be some local decision making, the collective consciousness has overarching control of the actions of the individual bodies. Goals and desires belong to the collective rather than to individuals. While pain or pleasure may be locatable in specific individuals, it is felt and understood as belonging to the whole. The relationship between the ‘individual’ aliens and the collective is something like the relationship between me and my thumb. When my thumb does something, *I* am the true agent of the action. When something acts on my thumb or does something bad to my thumb, it is the effect on *me* that is morally significant. Pain that is located in my thumb is *my* pain. Similarly, in the hive-mind case, it is the collective, not the individual aliens that act, are acted upon and have interests in a morally significant way. Such collective agency and interests cannot generate a morally significant connection between the individual alien and its body.^17^

In contrast, when a N–P&N–H-animal acts, suffers and has interests, in a morally significant way, as an individual, it seems they do have the kind of special connection to their body which requires us to recognise that body as belonging to them. I think that many N–P&N–H-animals, for example cows, do meet these conditions. I believe that a cow’s body is the locus of her morally significant individual agency: she, as an individual, can be seen as acting on the world in a morally significant way and does so through her body; it is the locus of her individual patience: the world acts on her, as an individual, through her body; it is the major locus of her morally significant individual interests: things can go well or badly for the cow, as an individual, in a morally significant way and what is happening to her body is a significant part of how well or badly things are going for her. Each of these statements is somewhat controversial and requires further argument.^18^ But if I am correct, this gives the cow a special connection to her body such that failure to recognise this special connection, treating the cow’s body as if it were an unallocated resource, fails to respect something important about the cow.

Nonetheless, there are important differences in the way in which a person’s body belongs to them and the way in which a cow’s body belongs to her. Respecting the way in which my body belongs to me involves respecting *both* my authority to make decisions about what happens to my body *and* my entitlement to make those decisions based on my own interests and desires. Respect for my authority to make decisions about my body is intimately connected to respect for my autonomy. If cows have autonomy, it is very different from the autonomy of persons. Similarly, if a moral code fails to recognise my special relationship to my body this means it does not give me appropriate moral standing, threatening to undermine my self-conception and my relationships with others. A cow’s self-conception cannot be undermined by a lack of moral standing. It may make sense to say that the cow’s relationships with some others are undermined because they do not appropriately respect her moral standing: for example, we might think that there is something deeply wrong in the relationship between a farmer and the cows he raises for food. But the threat to that cow-human relationship does not seem to be as morally significant a thing as it would be if my relationships with my fellow persons failed to recognise my moral standing.

Much of my second defence of the Body Claim does not apply to cows. Cows are not moral agents, and thus not subject to moral requirements. So normative imposition cannot undermine a cow’s Full-Fledged Agency. Nonetheless, the cow’s agency can certainly be threatened by others doing harm to her. However, again, it seems as if the agency that cows exercise is importantly different from the agency that persons can exercise. A threat to cow-agency may not be *as* morally significant as a threat to the agency of persons.^19^

All this suggests that we should accept a *weak* version of the Body Claim for N–P&N–H-Animals: for some N–P&N–H-animals, that animal’s body genuinely belongs to it, but the strength of a N–P&N–H-animals’ claims over its body are weaker than a person’s claim over their body. This leads to acceptance of a *weak* Full-Blown DDA for N-P&N–H-animals: there are constraints against doing harm to such animals, but these constraints are weaker than the constraints against doing harm to people.^20^

This seems to be not only the most theoretically defensible position, but also the view that has the most intuitively plausible implications for cases. Completely rejecting constraints against doing harm to N–P&N–H-animals gives peculiar results.^21^ It implies that it is permissible to kill one cow to save another simply because you prefer the colour of its coat. Of course, one might argue that this is wrong simply because it will make you yourself callous and cruel. But that seems to miss the point. To kill one cow to save another cow, surely wrongs the cow that you kill.^22^

Nonetheless, intuitively, the constraints against doing harm to N–P&N–H-animals are weaker than the constraints against doing harm to people. We may think it permissible to sacrifice one animal to save a greater number of other animals of the same type when we would not think it permissible to sacrifice one person to save the same number of people. For example, if a deadly disease is threatening a large population of giraffes, it seems permissible to deliberately infect a small number of giraffes with the disease in order to produce a vaccine. It is not intuitively permissible to deliberately infect people without their consent to produce vaccines.^23^, ^24^

Animals also interact differently with other factors such as species preservation and ecological diversity. For example: Goats are an invasive species in the Galapagos Islands, introduced by fishermen in the 1950s. The goat population rapidly spread and caused significant damage to the delicate ecosystem. Project Isabella, a multi-agency project launched in 1997, aimed to eradicate all goats, shooting them and leaving the bodies to rot to return the precious nutrients to the soil. Although Project Isabella involved slaughter on an immense scale, it is widely seen as acceptable.^25^

The ethics of Project Isabella are complex. The intervention may not be seen as a simple case of doing harm to the goats. If both the original fishermen and those taking part in Project Isabella are thought of as representatives of humanity, this can be seeing as a case of undoing harm ‘we’ ourselves have done or even as preventing ‘ourselves’ from doing harm.^26^ Moreover, goats, as a species, are portrayed as threats and the project is seen as defending both the famous Galapagos Tortoises and the ecological diversity of the islands from these threats. Nonetheless, at least part of this complexity is a result of treating constraints against doing harm to animals differently than we treat constraints against doing harm to people. Constraints against killing individual humans cannot be overridden by appeal to the threat posed by the human species–yet we treat constraints against killing goats as overridden by the threat posed by the goat species.

## Minimal and full-blown DDA and hybrid views of animal ethics

I now return to the hybrid approach to Animal Ethics discussed in the introduction: Utilitarianism for Animals; Deontology for People. What are the implications of the above discussion for this approach? The answer depends on what is meant by a hybrid approach to Animal Ethics and what is meant by Utilitarianism for Animals.

Nozick introduces ‘Utilitarianism for Animals, Kantianism for People’ by stating: “It says: (1) maximise the total happiness for all living beings; (2) place stringent side constraints on what one may do to human beings” (Nozick, 1974, 39).

Both the Minimal DDA and the Full-Blown DDA are clearly incompatible with this maximising act-utilitarian version of the Utilitarianism for Animals. Both versions of the DDA recognise permissions to allow harm to N–P&N–H-animals even when this is not optimific.

I think that the arguments in Section III and IV show that we should reject this version of Utilitarianism for Animals. First, this approach faces Hills’ Problem and thus has deeply counterintuitive results. Moreover, truly being Deontologists for People requires us to recognise the moral relevance of persons *both* as potential victims *and* as agents. If we allow the moral features of persons as potential victims to give rise to side-constraints on maximising utility, we should allow the moral features of persons as agents to give rise to permissions not to maximise utility.

It is better to understand Maximising Act-Utilitarianism for Animals as implying that N–P&N–H-animals themselves do not give rise to either constraints on maximising utility or permissions not to maximise utility. This allows us to recognise both deontological constraints that protect patients who are persons and deontological permissions that protect agents who are persons–and thus to be proper Deontologists for People. This view is compatible with the Minimal DDA, which appeals only to the need for permissions to allow harm to avoid normative imposition on the agent. It is thus able to avoid Hills’ Problem.

Of course, some might find this conclusion worrying. It seems odd to characterise as Act Utilitarianism for Animals a view on which doing harm to N–P&N–H-animals to avoid a certain cost may be impermissible while allowing harm to avoid the same cost would be permissible. A hallmark of Act Utilitarianism is usually that it holds that it is the consequences, not the agent’s relationship to the consequences, that matter.^27^ I am sympathetic to this reaction. However, if we insist that Utilitarianism for Animals means no difference between doing harm to N–P&N–H-animals and allowing harm to such animals, then the conclusion must be that Utilitarianism for Animals, Deontology for People is incoherent. Deontology for People must include permissions for agents to allow harm.

So the most coherent version of Maximising Act-Utilitarianism for Animals states that N–P&N–H-animals themselves do not give rise to either constraints on maximising utility or permissions not to maximise utility–but that people do. Although the implications of this approach do not exactly look Act-Utilitarian, it still deserves to be seen as an Act Utilitarianism for Animals and a genuine hybrid approach to animal ethics. It is genuinely Act-Utilitarianism for Animals because the only moral restrictions on our treatment of N–P&N–H-animals that come from N–P&N–H-animals themselves are considerations about how to maximise N–P&N–H-animal welfare. It is a genuine hybrid approach to animal ethics because people are treated strikingly different from N–P&N–H-animals in that they generate deontological constraints and permissions.

Nonetheless, I think we should also reject these versions of Act-Utilitarianism for Animals. I gave an outline argument above that (at least some) N–P&N–H-animals’ bodies do genuinely belong to them, in a way that gives rise to constraints on doing harm to them. If this argument works, we should reject the key idea of Act-Utilitarianism for Animals that N–P&N–H-animals themselves do not give rise to constraints on maximising utility.

Does this mean that we should reject all forms of Utilitarianism for Animals, Deontology for People? I noted in a footnote that Hills’ argument referred to Utilitarianism but does not apply to versions of indirect utilitarianism that endorse the doing/allowing distinction.Analogous versions of Indirect Utilitarianism for Animals may also be able to endorse a Full-Blown DDA for N–P&N–H-animals.^28^ I leave discussion of these for elsewhere.

## Conclusion

My aims in this paper were to unpick the implications of my defence of the DDA for animal ethics generally and for Nozickian hybrid approaches to animal ethics in particular. I also wanted to show that my account has the useful implication that we can avoid Hills’ Problem while making only minimal commitments about the moral status of animals. My focus was specifically on non-person, non-human animals (N–P&N–H-animals).

I’ve shown that my account allows us to defend a Minimal DDA by claiming (a) that the agents (who are persons) have authority over what belonging to them which requires permission to allow harm; (b) that the N–P&N–H-animal potential victim can be harmed in a morally relevant way which gives rise to a standing reason to avoid such harm. This Minimal DDA entails a moral distinction between doing and allowing which is enough to avoid Hills’ Problem.

For a Full-Blown DDA which recognises constraints against doing harm to N–P&N–H-animals, we need to make more substantial claims about N–P&N–H-animal moral status: we need to claim that (at least some) N–P&N–H-animals’ bodies belong to them in a morally relevant way. I argue that some aspects of my defence of the Body Claim for Persons transfer to many N–P&N–H-animals. Nonetheless, persons’ claims over their bodies seem to be stronger than the claims of such animals. This supports a Full-Blown DDA for N–P&N–H-animals which is weaker than the DDA for persons. The DDA for N–P&N–H-animals should include constraints against doing harm to such animals, but these constraints should be weaker than the constraints against doing harm to persons. This position also has the most plausible implications for particular cases.

The most plausible form of Act-Utilitarianism for Animals is the view that N–P&N–H-animals themselves do not give rise to either constraints on maximising utility or permissions not to maximise (or satisfice) utility–but that people do. This form of Act-Utilitarianism for Animals is compatible with the Minimal DDA and thus with my solution to Hills’ Problem. Nonetheless, if I am right that at least some N–P&N–H-animals’ bodies do belong to them in a way that gives rise to constraints against doing harm, we should reject even this form of Act-Utilitarianism.

I focus on my own defence of the DDA because how–and to what extent- the DDA applies to N–P&N–H-animals will depend on *why* the DDA applies to persons. Nonetheless, we can draw conclusions that are of wider interest. Any defence of the DDA that has the same structure as my defence will have structurally similar implications for N–P&N–H-animals–including the ability to avoid Hills’ Problem. A defence of the DDA has the same structure as mine if it recognises: (1) constraints against doing harm based on some feature of the potential victim; (2) permissions to allow harm based on some feature of the agent; (3) a reason to help creatures and prevent harm. Any such defence will recognise permissions to allow harm to animals and thus should be able to avoid Hills’ Problem with only minimal commitments about animal status. Whether each such defence recognises a Full Blown DDA with constraints against doing harm to N–P&N–H-animals–and how strong these constraints are–will depend on whether N–P&N–H-animals are taken to have the features that defence appeals to. Any defence which appeals to a feature that N–P&N–H-animals have to some degree, but not as fully as persons, will entail a weak Full Blown DDA for such animals.^29^

## Data Availability

Not applicable.

## Footnotes

1 Kittay (2008) criticises discussions that stipulate that the ‘congenitally severely mentally retarded’ lack the necessary capacities for personhood and shows the potential harm of hasty discussion of this issue.

2 I originally planned to simply use the term ‘animals’ and stipulate that this should be taken to mean non-person, non-human animals. Kittay (2008)’s discussion of the harms of such stipulations convinced me to avoid this approach.

3 I thank the anonymous referee who pressed me to clarify my approach to this issue.

4 Hills uses the term ‘Utilitarianism’ for her opponent’s view. However, Hills’ argument depends on the claim that Utilitarians do not recognise a morally relevant distinction between doing and allowing harm. Some versions of indirect forms of Utilitarianism can endorse the doing/allowing distinction. (See Woollard 2015, Chapter 9. See also e.g. Mill 1863, Chapter 5.) Hills’ argument does not apply to these versions of Indirect Utilitarianism. I thank Tyler John for pressing me on this.

5 Intervening to reduce the deaths of wild animals may lead to worse consequences overall. Hills argues that in this case, we are required to intervene to prevent fruitless suffering (Hills 2010, p. 236).

6 Hills makes this point (Hills 2010, 234), but the zebra example is my own.

7 I thank Anna Folland for pointing this out to me.

8 One might worry that even if we recognise a difference between doing and allowing harm, our duties to animals may still be very demanding given how much animal suffering there is. I do not think this is part of Hills’ Problem. Instead, it is a different problem which is the analogue of the problem raised by Peter Singer (1972) and Peter Unger (1996)’s arguments that given the extent of human suffering, we are morally required to give away most of our wealth to save those dying from poverty. My response to Singer and Unger’s arguments could be adapted to the animal case (2015, 121–171).

9 I thank the anonymous referee whose comments helped me make this section clearer.

10 If these reasons are weak enough, my solution to Hills’ Problem will not work. My solution needs the reasons against animal pain to be at least strong enough to rule out causing one hundred zebras to suffer the pain of being eaten alive in order to pursue my personal projects. I think fairly minimal assumptions about the moral significance of animal pain are enough to leave the standing concern for the good of creatures, unopposed by any permission to allow harm, strong enough for those purposes. I thank Adam Lerner for pressing me on this.

11 Kagan appeals here to what Regan (2004, 85) calls ‘preference autonomy’. See Regan (2004, pp. 84–86, drawing on Chapters 1 and 2) for an extended argument that animals have this kind of autonomy.

12 I thank the anonymous referee who pointed me to Healey and Pepper’s paper.

13 https://www.wptv.com/news/state/alligator-kills-florida-mans-service-dog I thank the anonymous referee who pressed me to make the example clearer.

14 This position conflicts with, for example, Carl Cohen’s view that “Animals are of such a kind that it is impossible for them, in principle, to give or withhold voluntary consent or to make a moral choice. What humans retain when disabled, animals have never had.” (Cohen 1986, 866) Healey and Pepper argue that animals are unable to consent but can give morally transformative assent (Healey & Pepper, 2020, 8–14).

15 For a classic defence of these claims, see Regan 2004, 1–81; 87–89.

16 To take just a few examples, Singer (1979); Cohen (1986), Regan (2004).

17 I thank the anonymous referee who pressed me to make these two cases clearer.

18 Regan (2004) provides a classic defence of the complexity of animal awareness and of the moral significance of animal’s agency and interests.

19 Regan (2004) argues that both humans and animals are subjects-of-a-life, that all subjects-of-a-life have equal inherent value which means we must not harm them in order to produce a greater aggregate benefit. It isn’t completely clear whether Regan’s harm principle should be read as prohibiting us from *doing* harm to subjects of a life or as acting in such a way that subjects of a life suffer harm. The fact that he recognises a separate ‘duty of assistance’ (xxvi) seems to make the natural reading a prohibition against doing harm. On this reading, Regan implicitly assumes the DDA without argument. There is a significant similarity between my criteria for giving rise to constraints against doing harm (acting, being acted on and having interests in a morally significant way as an individual) and Regan’s subject-of-a-life criteria for inherent value. From this point of view, my argument could be seen as filling a gap in Regan’s argument. But Regan would reject my conclusion that the constraints against doing harm to animals are weaker than constraints against doing harm to persons. I suspect he would accuse me of appealing to the consequences of respecting the subjects-of-a-life’s connection to their body, arguing that the requirement to respect this is independent of consequences (Regan 2004, 280). I do not take myself to be appealing to consequences when I note that e.g. a cow’s self-conception cannot be undermined by a lack of moral standing or say that it is more morally significant if my relationships with my fellow persons fails to recognise my moral standing. Instead, I take these to provide reasons that the connection between a person and their body is more morally significant than the connection between a cow and her body.

20 The Full-Blown versus Minimal distinction relates to whether all elements of the DDA are present i.e. both constraints against doing harm and permissions to allow harm. The weaker/ stronger distinction relates to how strong these constraints and permissions are. It is possible to have a strong Minimal DDA or a weak Full-Blown DDA. This may seem counterintuitive but I think these distinctions are useful. I thank Adam Lerner for raising this.

21 For similar argument see Nozick (1974, 40–42).

22 For similar argument see Nozick (1974, 41).

23 Shelly Kagan argues that holding that constraints against harming animals are as strong as constraints against harming imply that it is impermissible for a human castaway to kill and eat most animals in order to save the human’s life. (Kagan 2019, Chapter 7). I have concerns about Kagan’s calculations, so I use cases involving sacrificing one animal to save a greater number of animals of the same type.

24 My appeal to what is intuitively the case is not meant to be a claim about what most people would say in response to a survey. It should be understood more as an appeal to the reader to consider their judgments about particular cases which persist after reflection. If an account of ethics has implications which conflict with such judgments, the reader will find it hard to accept. For a persuasive account of this approach to ethics see Hooker 1996. Nonetheless, some interesting empirical evidence does suggest that the responses of many people to surveys show something like the pattern I endorse, see Kahane and Caviola (2015) in this volume. I take this to be helpful to my argument insofar as it suggests that many readers will be inclined to grant these premises.

25 I thank Clare Palmer for raising this case.

26 For discussion of letting oneself do harm see Persson 2013; Hanna 2015a, 2015b.

27 Killoren and Streiffer have a similar, but much longer, discussion exploring whether certain principles which could explain special obligations to animals are compatible with the commitments of Utilitarianism for Animals. They note that: “utilitarianism is usually understood to be an expression of the Moorean idea that the deontic supervenes on the evaluative, i.e., there can be no difference in the obligations faced by agents in two cases without a difference in the value of the consequences of the actions available to the agents” (Killoren & Streiffer 2018, 11). Utilitarianism for Animals might include a commitment to what they call *Restricted Supervenience*: the principle that there must be no difference in obligations in any two cases where the agent must decide whether to perform an action, performing the action affects animals but not people in morally significant ways, and there is no difference in the intrinsic value of the consequences of the action. The Minimal DDA is compatible with Killoren and Streiffer’s Restricted Supervenience principle, but conflicts with a suitably modified version of the principle which includes cases where the cost to the person agent of performing the action is equal and this cost to the agent is the only way in which the whether the action is performed affects persons in morally significant ways.

28 See Jeff Sebo’s paper in this issue for discussion of how utilitarianism for animals might be applied at different levels of moral theory.

29 See Kagan 2019, Chapters 8 and 9, for argument that any plausible candidate feature for deontological constraints should have this feature.
